# αβ T Cell Receptor Mechanosensing Forces out Serial Engagement

**DOI:** 10.1016/j.it.2018.05.005

**Published:** 2018-07-04

**Authors:** Yinnian Feng, Ellis L. Reinherz, Matthew J. Lang

**Affiliations:** 1 Department of Chemical and Biomolecular Engineering, Vanderbilt University, Nashville, TN 37235, USA; 2 Laboratory of Immunobiology and Department of Medical Oncology, Dana-Farber Cancer Institute, Boston, MA 02115, USA; 3 Department of Medicine, Harvard Medical School, Boston, MA 02115, USA; 4 Department of Molecular Physiology and Biophysics, Vanderbilt University School of Medicine, Nashville, TN 37235, USA

## Abstract

T lymphocytes use αβ T cell receptors (TCRs) to recognize sparse antigenic peptides bound to MHC molecules (pMHCs) arrayed on antigen-presenting cells (APCs). Contrary to conventional receptor–ligand associations exemplified by antigen-antibody interactions, forces play a crucial role in nonequilibrium mechanosensor-based T cell activation. Both T cell motility and local cytoskeleton machinery exert forces (i.e., generate loads) on TCR–pMHC bonds. We review biological features of the load-dependent activation process as revealed by optical tweezers single molecule/single cell and other biophysical measurements. The findings link pMHC-triggered TCRs to single cytoskeletal motors; define the importance of energized anisotropic (i.e., force direction dependent) activation; and characterize immunological synapse formation as digital, revealing no serial requirement. The emerging picture suggests new approaches for the monitoring and design of cytotoxic T lymphocyte (CTL)-based immunotherapy.

## Biophysical Mechanism of αβ TCR Triggering via an Energized Process

αβ T cells specifically recognize foreign peptides displayed on infected or otherwise perturbed cells through a process that discriminates with exquisite specificity. In so doing, T cells can discern a single amino acid difference between two antigens. At the heart of this process is a receptor–ligand interaction between variable domains on the αβ TCR and a peptide cradled in the groove of a major histocompatibility molecule, pMHC ([1,2] and references therein). APCs displaying peptides at single-molecule (SM) levels can be recognized by T cells [3,4]. Equilibrium between a bound and unbound receptor satisfies the law of mass action and mathematically relates the relative population of species found in the bound and unbound states; the ratio of the forward and reverse state transitions; or similarly the ratio of the state lifetimes. From an equilibrium perspective and our basic understanding of receptor–ligand associations, one expects high affinity. Paradoxically, however, TCR–pMHC affinities as conventionally measured by free-solution methods such as surface plasmon resonance reveal low affinity receptor–ligand interactions; typically in the low to high micromolar 3D affinity range.

Notwithstanding, the paradox that a mere handful of foreign peptides is sufficient for CTLs to mount a deadly response or helper T cells to activate despite apparent weak affinity was thought to be explained through a concept known as serial engagement [5]. Conceptualized 25 years ago, serial engagement (or serial triggering) recycles a single pMHC through multiple sequential TCR binding events to collectively stimulate a T cell over a time period (from seconds to hours) [6]. One ligand on an APC with intermediate affinity (K_D_ = ~1–5 μM) can thus activate in series a multiplicity of TCRs on a given T cell where the sum of integrated receptor activation collectively suffices to turn the T cell ‘on’ [7]. A fundamental limitation of this model is that it is not based on direct visualization and continuous measurement of the *in situ* dynamic interactions between TCRs and pMHCs at the live cell membrane. Instead it utilizes down regulation of TCR copy number as a parameter of TCR occupancy, assuming reutilization of one pMHC by many TCRs over a period of hours [8].

**Figure 1.**
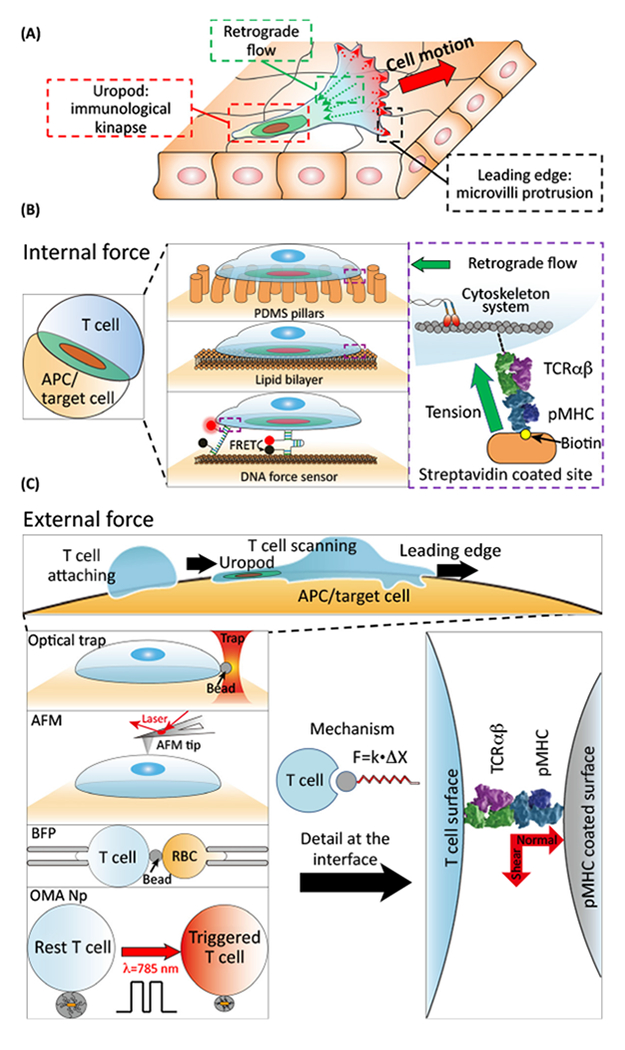
Characterization of Mechanical Forces Impacting T Cells. (A) Cartoon showing *in vivo* T ceil immunosurveillance (red arrow) along the surface of an epithelium or other cellular array. Note that substantial mechanical forces are exerted by protrusion of microvilli [50,51] (broken red arrows) at the leading edge and the retrograde flow (broken green arrows) due to cytoskeletal reorganization. The TCRs, initially localizing in microvilli, will be transported by retrograde actin flow to the uropod, forming an immunological kinapse with the APC. (B) Technologies for measuring and visualizing internal force generation during immune synapse maturation at the T cell–APC interacting surface. Traction force microscopy (PDMS pillar and lipid bilayer) and DNA force sensor are two typical methods. The detailed method descriptions are given in the Glossary. Such internal forces are mainly driven by retrograde flow through reorganization of cellular cytoskeleton, as highlighted in the purple box. (C) External force generated at the leading edge during T cell scanning APC/target cell surface can be imitated by optical trap, AFM, BFP, and OMA Np methods. All the methods are based on the spring-like features of the devices/material. With precise directional and distance control, optical traps are an ideal technology for testing the mechanosensing properties of the TCR onaT cell surface through a pMHC coated bead. Abbreviations: AFM, atomic force microscopy; APC, antigen-presenting cell; BFP, biomembrane force probe; OMA Np, Optomechanical actuator nanoparticle; PDMS, polydimethylsiloxane; pMHC, peptide bound to MHC molecule; RBC, red blood cell; TCR, T cell receptor.

T cell activation is an energized, nonequilibrium process. Physiologically, T cells patrolling lymph nodes or inflamed tissues are highly mobile [9]. The polarized cells exhibit directed motion with frequent stops and turns, where, from such global motions, the underlying adhesions experience local stress and directional physical force [10]. Cellular structures such as lamellipodia and **microvilli** at the leading edge will facilitate T cell activation [8,11,12] as well as a scanning trajectory that promotes local exploration of the microenvironment to find strong antigenic stimulation [13]. Later, T cells cluster their TCRs in the **uropod** (see Glossary), which is central to the cell and associated with actin **retrograde flow(RF)** [14,15]. Collectively, immuno surveillance-based cell crawling, microvilli protrusion and cytoskeletal movements can generate forces ranging from pN to nN [16–18], as shown in (Figure 1A).

After TCR activation, a structure known as an **immunological synapse (IS)** on stationary cell interaction or an **immunological kinapse** on motile cell interaction is formed [19]. Internal forces associated with these structures can be directly visualized by **traction force microscopy** (such as deformation of underlying elastic substrate or PDMS pillars) [20–22] or by signal changes in **tension gauge tethers (TGTs)** (such as DNA/peptide force sensors) that open under nominal threshold forces to emit a fluorescent signal (Figure 1B) [23]. Technologies for applying external force and monitoring local stiffness include **atomic force microscopy (AFM)** [24], **biomembrane force probe (BFP)** [25–27], **opto mechanical actuator nanoparticles** (**OMA Nps)** [28], and **optical trap/tweezers (OTs)** among others (Figure 1C) [29,30].

Such methods increasingly refine the link between TCR–pMHC binding and activation in a way to make probing serial engagement feasible. Using traction force microscopy, no evidence for serial engagement was found [31] but later invoked to explain an active feedback mechanism for early T cell activation that globally modulates TCR–pMHC binding [32]. In recent papers about BFP [27] and AFM [24], serial engagement, through repeat pulling on TCRs, is suggested to be required for T cell activation. Collectively, these advances failed to afford an unambiguous conclusion.

**Figure 2.**
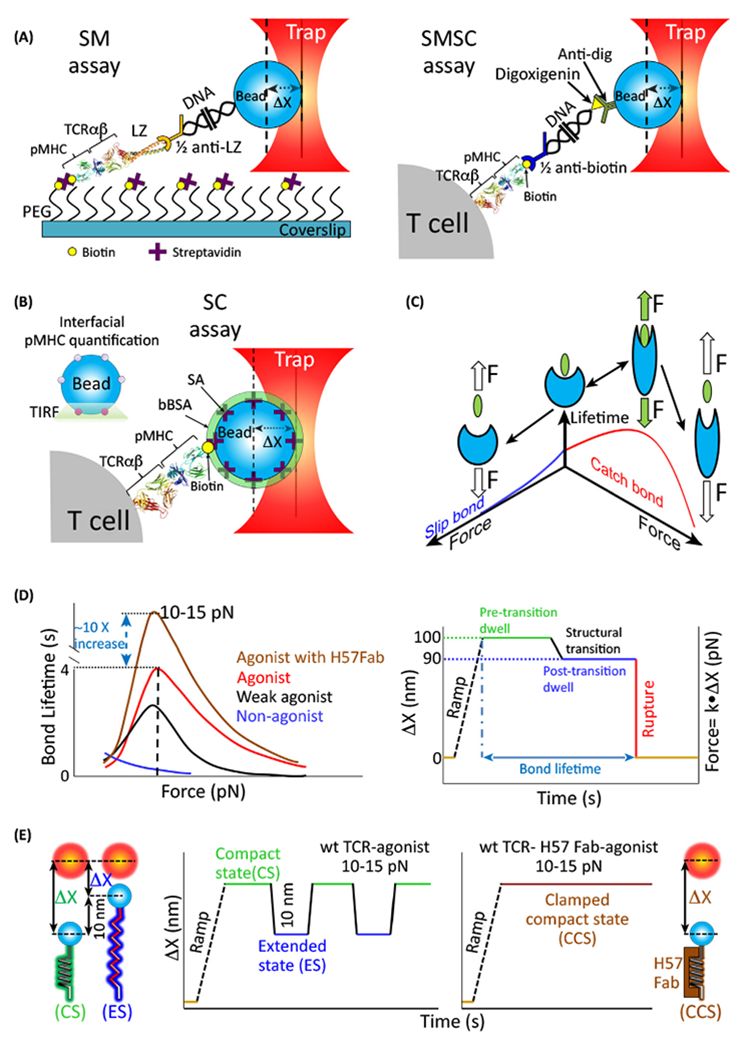
Mechanosensing Properties of the TCR Assessed by OT-Based Methods. (A) TCR mechanosensing is revealed by three OT assays. SM assays use a surface-attached pMHC and an LZ-fused αβ TCR to probe the lifetime of single αβ TCR–pMHC bonds. Biotin–pMHC molecules are anchored at the tip of PEG molecules through a biotin–streptavidin–biotin sandwich system. LZ-fused αβ TCR is linked to a half-2H11 (anti-LZ) antibody functionalized 1010-bp DNA at one end. The other end of the DNA covalently binds to a polystyrene bead surface. The SMSC assay reverses the SM architecture. AT cell expressing a specific TCR is attached on the coverslip surface. The biotin–pMHC is linked to a half anti-biotin antibody functionalized 3500-bp DNA at one end. The other end of DNA with dig tag binds to an anti-dig coated polystyrene bead. (B) The SC assay uses a pMHC-coated bead [made through biotin–streptavidin (SA) interaction] to bind the TCR on the T cell surface. The bead surface is then saturated with bBSA to prevent nonspecific binding. A pMHC-coated bead is placed on the waist of the surface attached T cell. Directional force (shear or normal) is generated by moving the stage to a certain displacement. Quantification of the interfacial pMHCs is performed by TIRF. (C) Aslip bond without the potential to allosterically change the receptor conformation is destabilized by applied force. By contrast, force exerted on an optimal ligand facilitates the receptor structural transition and αβ TCR ligand interfacial complementarity to deliver additional binding energy that stabilizes the bond thus creating a catch bond. (D) Catch bonds are observed for agonist (VSV8) and weak agonist (L4) but not nonagonist (SEV9) upon interaction with the N15 TCR, representative of those expressed on CD8 T cells specific for the vesicular stomatitis virus. Specifically, the TCR ligated by H57 Fab, an antibody that directly interacts with the FG loop of the TCR Cb region, increases catch bond lifetime ~10 times at optimal force. Bond lifetime is defined as shown, namely the time between the force ramp when force is applied and bond rupture. Structural transition (~10 nm at 15 pN) is visualized during the lifetime measurement as a molecular extension/alteration so that the bead begins to return to the OT center. ΔX denotes displacement of bead out of the center of the trap. (E) Reversible structural transitions of a single molecular αβ TCR–pMHC interaction are seen under 10–15 pN. This is depicted as the extension and releasing of a spring-like TCR under a trapping force as visualized in a single continuous recording. Strikingly, a clamped compact state is observed for an H57 Fab-clamped αβ TCR under the same force magnitude. Abbreviations: bBSA, biotin–BSA; CS, compact state; dig, digoxigenin; ES, extended state; LZ, leucinezipper; PEG, polyethylene glycol; pMHC, peptide bound to MHC molecule; SA, streptavidin; SMSC, single molecule on single cell; TCR, T cell receptor; TIRF, total internal fluorescence microscopy.

With increasing spatiotemporal resolution among the spectrum of SM force spectroscopy techniques [33] (in particular through OT-based methods [34], as shown in Figure 2A), direct testing of molecular association, structural transition, and bond lifetime of receptor–ligand interactions and characterization of binding, unbinding, and rebinding events is possible. An OT is formed by tightly focusing a laser beam typically to a diffraction-limited micron-sized volume [35]. If a tiny object such as a micron-sized plastic bead is near the focus, it is moved by light entering and exiting this region in a way that pulls it towards the central axis of the laser beam. OTs effectively represent a light-based ‘pick and place’ tool for manipulating small objects. If one moves the laser, one moves the trapped object. Using position-sensing systems akin to super-resolution methods, one can determine the center location of an object to nanometer level precision.

The manipulation ability of tweezers paired with the ability to directly visualize *in situ* dynamic interactions makes directly testing serial engagement mechanisms now feasible. Beads that mimic APCs are coated with pMHCs at known molecular densities. By directly labeling the pMHCs and using SM fluorescence imaging of these beads one can explicitly visualize the molecular density. Beads are then trapped and transported to a surface-bound cell where TCR–pMHC associations are actively facilitated. In Feng *et al.* [29], we systematically monitored the ability of T cells to activate with different numbers of pMHCs at the bead-cell interface, from high molecular densities (nonphysiological) to SM interactions. In addition to pick and place, binding and unbinding, an OT can be used to exert forces on an object. By measuring the displacement of the bead from the center of the trap (Δ*X*) and multiplying this by the spring constant of the trap *k*, the restoring force towards trap center is obtained (*Force* = *k*∙Δ*X*). The trap stiffness is measured straightforwardly and is typically expressed in units of pN/nm.

It is this force-exerting ability that revealed an alternative mechanism for TCR activation based on mechanosensing, as shown in the relevant experiment in Figure 2B. Pioneering work by Kim *et al.* demonstrated that T cell activation could be achieved when subjected to a low magnitude oscillatory force in the shear (tangential) but not normal (perpendicular) direction relative to the T cell surface [30]. Force was thought to push and pull on the TCR, permitting activation. Later, a type of binding interaction known as a **catch bond** was predicted [36] and subsequently confirmed with elegant BFP assays and OT-based measurements [27,37], as illustrated in Figure 2C,D, left. Conventionally, force accelerates bond release through an exponential dependence given by Bell [38] Catch bonds however exhibit an increase in lifetime in the presence of force, followed by a decrease at high forces. Direct measurement of the catch bond was pioneered by the Zhu laboratory using AFM and BFP; shown elegantly in systems such as P-selectin and its ligand [39], as well as many others. Mechanisms for such nonlinear bonding responses have been mathematically explained [40] and elaborated as force driven **allostery**, geometry and increased bonding at the receptor–ligand interface [41]. Catch bonds were observed for isolated SMTCR–pMHC interactions (Figure 2A, top left) and also directly on cells in an single molecule on single cell (SMSC) configuration (Figure 2A, top right) [37]. While catch bonds show an increase in lifetime, the mathematical effect on equilibrium based models is linear. Thus, it is not clear that the increase in lifetime from catch bonds alone, which may be tenfold, can explain the observed increase in sensitivity and selectivity among ligands, which can be many orders of magnitude [42].

In OT studies, another clue arose. A structural transition was observed for the αβ TCR both in SM and SMSC assays (Figure 2D, right), manifest as an extension of the molecule under force, thus reducing the displacement observed (Δ*X*). This transition not only requires work done on the TCR–pMHC bond, but it correlates with the presence of a catch bond state [43]. The structural transition likely alters the loading pathway, changing the distribution of force sustained through each bond. It may also allosterically alter the bonding interface between the TCR and pMHC (Figure 2C). Furthermore, as shown schematically in Figure 2E, overtime the loaded TCR reversibly transitions between compact and extended states (CSs and ESs, respectively). Structural transitions may also impact larger assemblies including that of the TCR and its co-receptor working in tandem during a pMHC interaction [44]. Of note, H57 Fab, an antibody fragment binding to the FG loop of the β TCR constant region, generally blocks the primary transition (and signaling), constraining the TCR ina compact state, compared to the multiple reversible transitions observed for wild-type αβ TCR. This force-based (energized) mechanism is by definition distinct from purely equilibrium binding mechanisms, as compared head to head in Feng *et al.* [29].

Additional studies have tied external force to activation. These include: Li *et al.* in which a micropipette and a shear force were associated with activation [26]; Husson *et al.* where pushing and pulling with a BFP activated cells [25]; Hu and Butte where T cells were triggered by a pMHC-functionalized AFM tip [24]; Liu *et al.* where catch bonds were observed and repeat pulling on the apical tip of T cells activated them [27]; and Liu *et al.* where OMA Nps were used to stretch cell surfaces, leading to activation [28]. Here we outline results involving mechanical measurements. These findings reveal that force enhances the sensitivity of T cell activation and that the direction of force differentially distributes load on individual TCRs, impacting the signaling requirements of pMHC copy number on APCs (Box 1).

## Regular Steps Linked to Actomyosin Machinery Rather than TCR Serial Engagement

**Figure 3.**
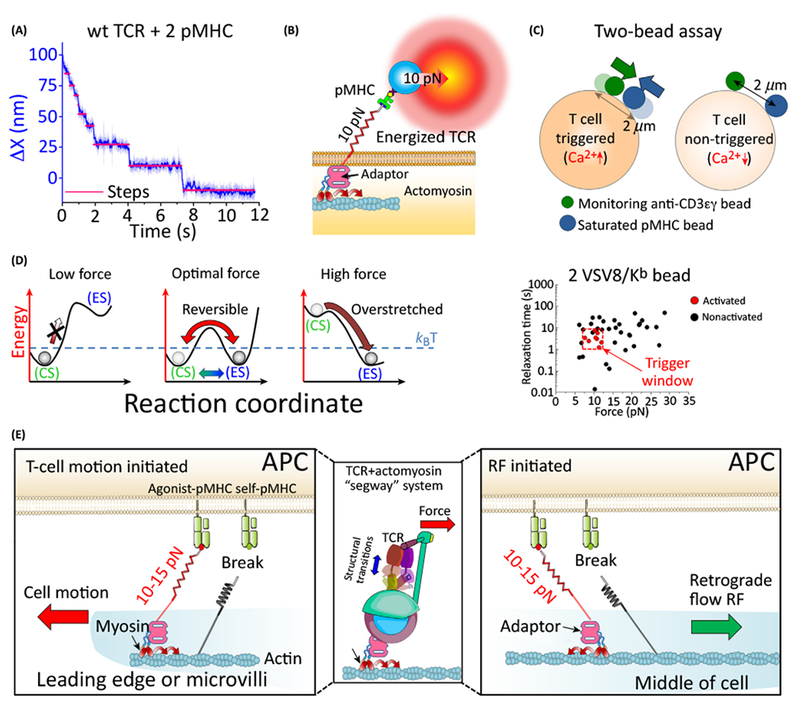
SC Experiments along the Shear Direction Reveal the Biophysical Reversible Transition Mechanism of TCR Mechanosensing. (A) wt TCR interacting with two interfacial pMHC shows a stepwise trace as retrograde flow moves the bead back to the OT center. The dwells are separated by ~8-nm steps with 100–400-ms intervals. (B) TCR mechanosensing system includes the spring-like αβ TCR and underlying actomyosin connected by an adaptor whose molecular definition is yet to be defined [76]. (C) Mechanical force loaded through motion of saturated pMHC-coated bead is the initiator of immune synapse formation in this experimental system, as pMHC-triggered T cells cluster ligand-engaged as well as pMHC-unengaged TCRs (the nonactivating 17A2 anti-CD3δγ mAb monitoring bead). Note that both beads are stagnant for nontriggered T cells. (D) An equilibrium process with thermal energy alone (left RC) is not sufficient to bypass the barrier. Optimal force tilts the landscape permitting reversible structural transition between ES and CS (middle RC). Excessive force constrains the system in the ES conformation with no reversible hopping (right RC). Relative energies are not to scale but rather show trends associated with tilting of the reaction coordinate under load. Plot of relaxation time versus force reveals a sweet spot for T cell triggering (red box). Relaxation is due to movement of the bead back to theOT center, a self-balancing process involving OT force and cytoskeletal motor. The feedback is akin to a self-balancing ‘Segway’ system. (E) Possible biophysical mechanism of TCR mechanosensing under physiological conditions. At the leading edge, cell motion will generate tension on the TCR–pMHC bond (left), and at the uropod tension is mainly generated by RF (right) where myosin stepping on F-actin sustains the optimal force, akin to our SC model in (B). At this optimal force, multiple, reversible structural transitions of TCR–pMHC system are possible, driving activation of the T cell. The potential linkage may be actin-myosin IIA-β arrestin-TCR complex [57,77,78]. Abbreviations: CS, compact state; ES, extended state; OT optical trap; pMHC, peptide bound to MHC molecule; RC, reaction coordinate; RF, retrograde flow; TCR, T cell receptor; wt, wild type.

Cell motions derive from underlying cytoskeletal elements and associated motor proteins. The major structural cytoskeletal elements are polarized filaments of actin and microtubules and their associated motors including myosins and kinesins plus dyneins, respectively [52]. Motion and forces can originate directly from these structures through polymerization and depolymerization processes and directed flow associated with building and recycling the cytoskeletal elements. Displacement can also arise from direct motion of motors walking on their cytoskeletal tracks. SM assays have reconstituted motility in isolated assays revealing direct stepping, length of stepping (or processivity), and **stall force** and rupture properties of the individual motors [53]. A range of cytoskeletal- and motor-disrupting drugs are available to test the origins of such motion. In fact, vigorous movements during early activation prior to IS formation has been observed by many groups [21,24,54–56], also including by visualization through lattice light sheet microscopy [15]. Actin polymerization and non-muscle myosin IIA contraction have been found to drive a rapid inward translocation of TCR microclusters only during the early stage of signaling due to the potential transient linkage between TCR microclusters to the underlying centripetal actin flow in the distal super molecular activation complex (dSMAC) [57,58]. At later activation stages, TCR microclusters are continuously moving inward across the proximal SMAC (pSMAC) with the help of being swept between adjacent actomyosin arcs at a slower velocity than that in dSMAC [59–61 ]. Compared to the SM tracking of internal force, active external force measurement on T cells requires firmly attaching the cell to fix its frame of reference relative to the force probe. The goal is to create a system that mimics a T cell crawling on a surface while permitting spatiotemporal sensing of these tools to interrogate the mechanobiology involved. With a T cell firmly attached on the coverslip, or held in a micropipette, one can monitor bonding events underpinning TCR activation. The super-resolution capabilities of tweezers position sensing has revealed that TCR–pMHC bonding is associated with active motor-based transport. In addition, traces show discrete steps (Figure 3A). One might argue from a serial engagement perspective that these are dwells between hops among different TCR molecules. We argue here that this is not the case. Rather, several observations support that the dwells are a result of motor-based transport [29], as illustrated in Figure 3B. First, the steps are regularly spaced, suggesting motility is on an underlying track or lattice of actin microfilaments or microtubules. Bonding is persistent. By contrast, if unbinding or rebinding events were mediated via a serial engagement mechanism, those would produce dwell locations at random positions manifest as irregularly sized steps. Second, the steps disappear when the cell is pretreated with myosin- or actin-disrupting drugs. Third, in the presence of H57 Fab, an anti body fragment ligating the Cβ FG loop [62] shown to sustain TCR–pMHC bond lifetime [37], identical stepping profiles are observed. In the presence of H57, TCR–pMHC single bond unbinding is virtually eliminated or severely reduced, yet similar step profiles are seen. A number of other observations clearly show that this structure is motor based. Moreover, we were able to measure a stall force and to demonstrate motion against the trap consistent with molecular motor function. Correspondingly, for force-free experiments described above, activation using high numbers of pMHC molecules (2 × 10^4^) per bead also showed displacement for triggered cells through internal cell cytoskeletal motions [29].

## Implications of Triggering Seen at Very Low pMHC Number

With a mere two molecules at the interface, we can directly visualize binding and unbinding events. Both lowering the pMHC concentration on the bead and pulling it away from the T cell surface should reduce any rebinding probability for serial engagement mechanisms. Yet, a higher probability of triggering was seen with normal pulls than tangentially applied forces with an extreme 2 pMHC molecules/interface (Box 1). From a mechanosensor perspective, lowering the pMHC concentration at the solid bead surface increases the distributed force per TCR–pMHC bond, increasing the probability that the system will be mechanically activated (structurally transitioned). In the native system, T cells crawl on the APC surface where increased peptide dose leads to increased activation. Local microvillus protrusions at the interface can efficiently mechanically activate many TCR–pMHC pairs producing large signature signals characteristic of these systems [47]. In the SM system, rather than discrete bound and unbound states expected for serial engagement mechanisms, we observed sustained attachment of TCR–pMHC bonds under load. At the SM level (0.5 pMHC/interface), the abrupt bond rupture between single TCR–pMHC interaction permits bead snap-back to the trap center. Such immediate bond breaking without productive serial turnover was also seen when using a weak agonist pMHC (L4/K^b^) forN15 TCR and high coating density close to the totality of self-pMHC molecules on an APC. L4 differs at only the p4 residue from VSV8, the cognate ligand for the N15 TCR, but manifests 10 000-fold lower **functional avidity** than the strong agonist VSV8 [42]. The stimulation of TCRs by L4/K^b^ was only observed at nonphysiologically high levels and with the help of external force application. Perhaps this weak interaction is equivalent to that mediating tonic TCR-self pMHC stimulation important for homeostatic proliferation [63]. These results cast doubt on the necessity of serial engagement in early T cell activation, even when weaker ligands are involved, contrary to the conventional serial engagement predictions [5]. Furthermore, the calcium flux induced here (~3.5 min) by force-facilitated TCR triggering suggests they are sufficient for inducing downstream biological outcomes. It has been shown that with ~90 s calcium flux a CD4^+^ T cell can induce interleukin-2 production by a single pMHC molecule [4], whereas CD8^+^ T cells require ~3 pMHCs with ~20% calcium increment to induce cytotoxic function [3].

## TCR Mechanosensing Initiates Formation of ISs

TCR activation must deal with a broad range of APC surface topologies arraying pMHC molecules. An outstanding question is whether IS formation is an artifact of the assay confined to a 2D planar surface or a geometry required for subsequent TCR activation. The two-bead experiment with one bead containing pMHC molecules and a second, nontrapped bead coated with a nonactivating anti-CD3 monoclonal antibody (mAb) provides direct evidence that force is the initiator of the IS (Figure 3C). During the internal force-induced activation by the first bead displaying 2 × 10^4^ interfacial pMHC molecules, pMHC-unligated TCRs detected by the second bead are recruited into the activation site, initiating formation of the IS. The initial force-induced TCR binding thus may drive intracellular signals to locally activate the actin cytoskeleton. Combined with the active transport exhibited in force-induced activation, non-triggered TCRs are recruited by motor-driven coupling during the maturation of IS. Note this process occurs in seconds, consistent with recent super-resolution data for CTls [15]

## Nonequilibrium Mechanosensing Activation Provides a Sensitivity Gain Factor Consistent with αβ TCR Performance

Equilibrium and nonequilibrium processes relate to serial engagement and mechanosensing mechanisms, respectively. Energetically, an equilibrium process is driven by thermal fluctuations (thermal energy *k*_B_T = 4.3 pN∙nm) where the system can freely move between states and the relative lifetimes of bound and unbound states (or equivalently population of states or ratio of forward and reverse rates) determine the distribution through a simple mathematical ratio. By contrast, what matters in a nonequilibrium system is whether a process has the required energy to surmount a barrier (Figure 3D, left). Instead of relating proportionally to lifetime, this probability of transiting a barrier is exponentially related to energy driving the process (i.e., Arrhenius activation energy). How much energy is associated with TCR–pMHC mechanosensing? Work with units of energy is the product of force and displacement in the direction of the force. The TCR–pMHC conformational change is on the order of 10 nm with a force of ~15 pN representing ~150 pN∙nm of work. If we relate this to thermal energy (*k*_B_T) this is 37 *k*_B_T, which is greater than the energy available from thermal fluctuations of the system. In fact, it is close to twice the energy gained from hydrolysis of ATP (1 ATP = ~20 *k*_B_T). Under equilibrium, we expect ~*k*_B_T worth of energy to be driving such a process. Thermal fluctuations can impart energy, but, based on these calculations, not nearly enough energy to drive the transition commensurate with observed T cell sensitivity, which has been found to be 1000–10 000 times better against its agonist versus an endogenous peptide [42,64]. Under a nonequilibrium process, the reaction rate is linked to the Arrhenius equation, in which the ratio of available energy to k_B_T exponentially impacts the reaction rate value. If a quarter of the work energy exchanged from a single transition is transduced (through an activation barrier) into the T cell, the gain is massive, 10 000-fold or ~4 orders of magnitude relative to an equilibrium driven process. Although catch bonds create an increase in lifetime, this factor (which may be 10 x) will only shift the equilibrium by a similar factor, which is not enough to explain the huge sensitivity disparity seen among peptide discrimination, which can be explained through nonequilibrium activation. It has been noted that the conformational change is reversible, providing multiple opportunities to deliver energy for activation. The window for triggering T cells shows that a force close to this threshold for conformational change is required for activation (Figure 3D, right). We have observed if one hovers near the critical force, reversible transitions are possible. In the original anisotropic mechanosensing studies, an oscillatory force was applied that may have created a scenario where multiple transitions were possible [30]. BFP assays require repeat pulling for activation [27]. AFM experiments in the absence of actin show triggering with oscillatory motion of the probe tip [24]. The TCR appears to derive power from the cellular grid. Allowing for repeat conformational change may be an advantage in activating TCR complex components.

## Force Feedback Maintains the TCR–pMHC Bond in a Sweet Spot Where Consecutive Conformational Change Occurs

The relative position of the T cell, including underlying cell movement during tissue scanning and its surface αβ TCR with respect to pMHC on the APC determine how fast the bond is loaded (Figure 3E). Thus, presence of a robust actin cytoskeleton before IS formation is expected, especially for a cell undergoing surveillance, as was observed by the recent lattice light sheet microscopy [15]. Interaction between the TCR and F-actin in this early state provides the substratum for mechanically actuating the TCR-motor-actin system [57]. It is interesting that cells that trigger show motor-driven displacement of the bond parallel to the pull direction such that tension is reduced (Figure 3B,E). This motion effectively decelerates the loading rate and maintains the bond in the sweet spot (near critical force) for conformational change akin to the control system in a ‘Segway’ (Figure 3E, middle) once the desired balance (force threshold) is reached. Force feedback may be important for facilitating signal activation by amplifying the amount of energy delivered to the system by controlling the amount of time that the TCR–pMHC bond is near the critical force. Consistent with this notion, a recent AFM publication by Butte’s group also gives a hint for the sweet spot [24], in which the elasticity of the dynamic cell membrane may maintain force in the sweet spot [65].

## TCR Complex Reconfigures through a Process Akin to Phase Transition

These experiments reveal a nonequilibrium metamorphosis of the TCR upon activation. In this regard, the αβ TCR is a squat but wide multisubunit protein complex composed of a disulfide-linked αβ TCR heterodimer flanked by three sets of noncovalently associated dimeric CD3 subunits: the CD3εγ and the CD3εδ heterodimers and the CD3ζζ homodimer [1,66–68]. Energizing this receptor system transforms the well-organized complex topology to one that extends the centrally disposed αβ TCR heterodimer, mandating additional force-directed alterations of CD3 dimers. Such rearrangements may trigger new associations as well as dissociations including with negatively charged vicinal phospholipids, and as a consequence, releasing positively charged tail segments of CD3 molecules to expose immunoreceptor tyrosine-based activation motif (ITAM) for phosphorylation [69,70]. Successive extension and retraction of the αβ TCR as observed in our single αβ TCR–pMHC traces [43] will further energize the complex and lipid bilayer to mandate additional conversions much like an agitator in a washing machine. Given interdigitation of juxtamembrane segments and interacting of transmembrane segments and surrounding lipids, it is inescapable that force will transduce biochemical changes from ecto domains through linkers, transmembrane segments, and the cytoplasmic tails (reviewed in [69]). Such blossoming changes in the organization are akin to a phase transition where a solid-like well-organized crystalline system is fluidized and reconfigured as mechanical programs drive formation of structures such as the IS. Phase transitions are associated with exchange of energy. Moreover, the underlying kinase pathways can further amplify the initiated mechanical signaling via their phosphorylation cascades.

## Comparison of αβ TCR Mechanosensing with Prior Models of TCR-Mediated Activation

Since the T cell signaling cascade begins with TCR–pMHC ligation, myriad TCR triggering models have been proposed over the years. These include the serial engagement model noted above, TCR oligomerization/aggregation, TCR conformational change models, kinetic proofreading concepts including co-receptor synergy, signaling amplification models involving endogenous pMHC, and the kinetic segregation model (reviewed in [71,72]). While artificial aggregation of TCRs by pMHC tetramer or antibody is sufficient for triggering the T cell activation cascade, the frequently low density of foreign pMHCs on infected or transformed cells ([73] and refs therein) casts doubt on the physiological relevance of massive clustering. Crystallography studies to date have provided some evidence for discrete TCR conformational change upon pMHC ligation, but those data are confounded by the potential for crystal lattice artifacts [71]. Moreover, crystal structures are generated in the absence of physical load, and therefore unable to reveal the key transitions required for catch bond formation involving dynamic conformations that are force driven [43]. Kinetic proofreading as well as signal amplification by endogenous pMHC models are suggested based on off-rate difference between agonist-pMHC and self-pMHC measured by surface plasmon resonance under force-free conditions. With respect to kinetic proofreading, the notion is that small differences among receptor–ligand pairs can be amplified differentially by several downstream components in a signaling pathway. However, recent OT and BFP experiments clearly show the enormous amplification of such minor differences through mechanical force application in a range observed physiologically [27,37]. The importance of nonequilibrium binding for T cell function is exemplified by elegant studies involving design of superphysiological (i.e., very strong) TCR affinity under zero force that nonetheless paradoxically yielded reduced functional T cell activation [7]. Mutations in complementarity determining regions (CDRs) that create the αβ TCR superphysiological binding at zero force could readily impair αβ TCR conformational change under nonequilibrium binding that is important for biological recognition of antigen fostering downstream signaling.

Kinetic segregation has been widely invoked in consideration of T cell activation and has also been observed in a reconstituted system [74]. Within the IS, the large ectodomain of the CD45 phosphatase relegates it to the dSMAC separated from the central SMAC (cSMAC) where TCR and pMHC and kinases such as Lck come to reside. This process can be modulated by genetically modifying the molecular size of SMAC proteins [75]. However, elongation of pMHC on the APC can disadvantage physical force generated from T cell crawling and/or retrograde flow, hindering T cell activation. Importantly, kinetic segregation cannot explain why a transgenic TCR with a CβFG loop deletion expressed at similar copy number to a wild-type TCR fails to negatively select thymocytes *in vivo* or trigger mature T cells unless, in the latter case, antigen concentration is increased by orders of magnitude. Collectively, these data suggest that kinetic segregation is a means to amplify TCR triggering rather than to initiate it.

## Concluding Remarks

Elucidation of T cell mechanobiology principles makes it clear that the targeting of viral or tumor-specific antigens need not exclude candidates expressed at relatively low copy number per cell, assuming potent αβ TCRs are elicited via vaccination or arise naturally. Likewise, there is no requirement for TCRs with a fast off-rate to foster serial engagement. As physical force tunes αβ TCR recognition acuity, TCRs manifesting 1–10-s bond lifetimes generally are efficacious to foster TCR activation. That said, the ligand binding approach vector is important in the force transduction process [29,30], likely linked to requisite conformational changes in the TCR complex ectodomains and transfer to transmembrane and cytoplasmic domains coordinated with changes in vicinal lipids. Immunotherapeutics based upon native αβ TCR as well as chimeric antigen receptor transduction into autologous T cells can be examined by OT methods described herein for optimization of ligand triggering. Moreover, with regard to immune monitoring, given that ELIspot and tetramer technologies bypass external force application to assess the quality of the TCR–pMHC bond, it is not surprising that such assays may fail to identify key biomarkers of clinical outcome and/or vaccine responsiveness. When ELI spot and related methods are used to quantify cytokine production, stimulation by antigen generally uses micromolar concentrations of peptide. This concentration is well above the physiological range and fosters TCR cross reactivity that is less likely to be observed at peptide concentrations in the nanomolar to picomolar ranges [67]. While there are multiple outstanding questions remaining to be answered (see Outstanding Questions), implementation of mechanobiology principles in assessing T cell adaptive recognition should be a game changer in the field.

## Highlights

The αβ TCR is a mechanosensor whose force-dependent structural transition and allostery regulate peptide discrimination and pMHC bond lifetime.

Application of mechanical force on the TCR during ligand recognition promotes its molecular translocation and initiates T cell immunological synapse formation.

Synergy of external (cell motility based) and internal (cytoskeletal motor based) forces supports a nonequilibrium (energized) model for T cell activation through reconfiguration of the αβ TCR complex at a critical force threshold.

A digital mechanosensing mechanism defines physicochemical thresholds with significant implications for CTL-based vaccines and immunotherapies. That knowledge affords new insights relative to earlier αβ TCR activation models based on equilibrium processes.

## Box 1. Internally Generated Force, Externally Applied Force and Direction-Based T Cell Activation

### Internally Generated Force Activation

Activation occurring passively through forces native to the cell generally refers to the methods shown in Figure 1B in main text, where forces arising from internal cell cytoskeleton motions activate T cells. Such forces are monitored and quantified by force sensors on the coverslip surface [45] or deflection of pillars [20,21]. However, higher local pMHC density and nN force magnitudes typical of these assays have masked direct elucidation of the molecular associations underpinning activation. Estimates based on the total force and local pMHC density suggest each TCR sustains tens of pN. This force threshold (10–20 pN/TCR) for T cell activation has been observed in the recent publications by the molecular tension fluorescence microscopy [23]. In OT experiments without external applied force, activation requires higher local pMHC density and exhibits large bead motion due to the transport via cytoskeletal machinery where internal forces are likely contributing to activation.

### Externally Applied Force-Based Activation

By contrast, force can be applied actively. There are two classes of large forces applied over many molecules and precision forces applied through a few or individual molecules. External forces can be applied to probe the triggering mechanism of individual cells or single receptor–ligand complexes, serially, one bond at a time. With force applied through the OT at 10–20 pN/TCR, cells are able to be activated with as few as two TCR–pMHC interactions at the interface [29]. This is strong evidence that the TCR harnesses a force-based mechanism for triggering.

### Direction-Based Activation

AT cell scanning an APC features highly organized cytoskeletal structures such as leading edge, uropod, and microvilli. Measurements of activation have largely focused on force applied normal or in shear directions relative to the generally spherical cell surface. How these forces interface with the underlying microstructure and distribute their loads across bonded TCRs impacts activation. The direction of force application significantly impacts cell functions [46]. Microvilli present on the T cell surface may apply shear force when scanning the APC [11,47], facilitating TCR triggering. In OT experiments on single cells a preference for force applied in the shear direction versus normal direction was observed. In these assays the pMHC-coated bead is placed at the waist of the T cell, far away from the cell coverslip adhesion site where abundant microvilli exist [11,48]. Normal forces activate more readily with fewer pMHCs at the interface, although shear direction favors more sustained Ca^2+^ flux. The specific pulling vector on the TCR–pMHC bond must take into account the geometry relative to the cell surface microstructure and likely includes some contribution from both normal and shear components, even in OT experiments where the bead can rotate and weak forces are utilized. In other methods such as AFM and BFP where force magnitudes capable of distorting the T cell are applied, stretch in axes orthogonal to the pulling or pushing directions occurs, and additional care is needed when interpreting direction with respect to both the cell probe frame of reference and microstructure presentation of the TCR–pMHC bond.AT cell held in a micropipette displays abundant microvilli [49]. BFP assays pulling normal with respect to the T cell will inherently apply shear motions along the microvilli. In AFM experiments pushing may apply more shear along the tip needle in these cases so that the correlation between the Ca^2+^ flux and the force magnitudes is obscured [24].

## Outstanding Questions

Is the TCR–pMHC bond lifetime force curve a key indicator of TCR quality linked to protective biology as opposed to conventionally measured functional avidity or tetramer staining?

From the molecular perspective, how does force-dependent transition in the αβ TCR heterodimer influence the quaternary structure of the αβ TCR complex and conformations of the CD3 cytoplasmic domains to initiate signaling?

What is the structure of the extended and force-loaded relative to the compact and unloaded αβ TCR complex?

Can adhesion molecules stabilize a single TCR–pMHC bond to facilitate T cell activation in a digital manner?

What is the precise molecular identification of the biochemistry, physiology, and associated protein interactions of the motor protein activated by TCR–pMHC bond formation?

On the APC side, how does viscoelasticity of the pMHC embedded in the membrane affect the TCR mechanosensing and force-induced T cell activation? Is this altered when dendritic cells go from an immature to a mature state?

How does the mechanical force applied on the TCR–pMHC interface initiate the actomyosin activity?

## Glossary

Allostery: protein structural change induced by binding of a substrate or other effector at a site (allosteric site) other than the chemically active site of the protein.
Atomic force microscopy (AFM): a form of scanning probe microscopy where a cantilever with a tip is systematically scanned and poked onto a sample (biological) to produce deflections that measure forces between the tip and sample. AFM’s work in the range of 10 pN–100 nN.
Biomembrane force probe (BFP): a technique that uses deformation of a membrane structure such as a lipid vesicle or red blood cell (RBC) under tension as a sensitive and adjustable force sensor. The RBCs are usually functionalized (e.g., biotinylation), with a stimulatory/interacting bead at one end and a suction micropipette at the other end. The measured forces can be ranging from 0.01 pN to 1 nN.
Catch bond: a bond for which lifetime increases with applied force up to a maximum, optimal force, and then decreases with greater applied force.
Functional avidity: T cell activation threshold measured as the concentration of peptide ligand required to trigger cytokine production, cytolytic activity, and/or proliferation of monoclonal or polyclonal T lymphocytes. It is thought to reflect affinity of the TCR for pMHC, expression levels of TCRs and co-receptors and cellular distribution and quantities of signaling molecules associated with a T cell.
Immunological kinapse: an unsymmetrical pattern akin to IS generated in a mobile T cell-APC contact during immunosurveillance, leading to T cell activation.
Immunological synapse (IS): a cytoskeletal structure resembling a bull’s eye forms at the interface between a lymphocyte and a pMHC surface array usually in an experimental schema such as a glass supported planar lipid bilayer. Typically, with the IS there is formation of a central TCR–pMHC cluster (cSMAC) surrounded by a ring of adhesion molecules (pSMAC) and a distal ring (dSMAC) that includes the activation inhibitor. This structural is essential in lymphocyte activation, cytokines secretion.
Microvilli: finger-shaped membrane protrusions about 140–380 nm in length, and 50–90 nm in diameter, with a core of parallel bundled actin that are involved in a wide variety of cellular functions, including cellular adhesion and mechanotransduction.
Optical trap/tweezers (OTs): a form of probe microscopy where a tightly focused laser beam is used to manipulate μm sized particles such as plastic beads. Forces in the range of 1–100 pN can be applied with exceptional position resolution, the properties of the optical trap for small displacements (~200 nm) are spring-like or Hookean. When a trapped bead binds to an object the distribution of fluctuations (Brownian motion) changes. Thus, one can observe the width of the distribution to track molecular binding and unbinding events.
Optomechanical actuator nanoparticle (OMA Np): a nanoparticle that grafts ligand functionalized thermosensitive polymer on a gold nanoparticle. Illumination with near-infrared light heats the gold nanoparticle, which leads the polymer coating to collapse, delivering pN forces to specific cell surface receptors with high spatial and temporal resolution.
Retrograde flow (RF): also named as centripetal flow, is the collective movement of actin filaments generally in a direction opposite to the movement of the cell or to the center of the attached cell. Both actin polymerization and myosin motors are crucial in driving retrograde flow.
Stall force: the force required to stop the translocation of an individual cytoskeleton motor along its trail. This condition occurs when the load is equal to the maximum force motor can exert.
Tension gauge tethers (TGTs): a method using short DNA duplexes or peptide as force reporters based on their specific sequences. The designed sequences define general tension tolerance ranges and will separate if tension above their tolerance is encountered. Unquenching of fluorophore is used as readout. When placed within a loading path, this method is useful for *in situ* visualization of single molecule pulling events by fluorescence microscopy.
Traction force microscopy: a method for visualizing forces cells exert on surfaces through distortions of a deformable material. The probes include fiducial marks such as fluorescent beads or micropillars made of elastic materials. Precise forces can be calculated by calibrating the mechanical properties of the material or micropillar beams..
Uropod: a rear part of polarized, motile cells crucial to maintaining cell migration. For lymphocytes, immunological kinapses (dynamic IS) are usually found in this region.
